# Human papillomavirus vaccination at the national and provincial levels in China: a cost-effectiveness analysis using the PRIME model

**DOI:** 10.1186/s12889-022-13056-5

**Published:** 2022-04-18

**Authors:** Liangru Zhou, Baiyang Gu, Jian Wang, Guoxiang Liu, Xin Zhang

**Affiliations:** School of Health Management, Harbin Medical University, 157 Baojian Road, Nangang District, Harbin, China

**Keywords:** Human papillomavirus vaccine, Cervical cancer, China, Health economics, Disability-adjusted life years

## Abstract

**Background:**

Human papillomavirus (HPV) vaccines have been proven effective against cervical cancer. However, HPV vaccination is not included in the Chinese immunization program. This study aimed to assess the cost-effectiveness of incorporating different HPV vaccines into immunization programs at the Chinese national and provincial levels.

**Methods:**

The Papillomavirus Rapid Interface for Modeling and Economics model was used to estimate the possible cost and social and economic benefits of adopting various HPV vaccination immunization strategies in 31 provinces in Mainland China in 2019. Demographic and regional economic data were obtained from the national and provincial Statistical Yearbook. The cost of vaccines was gathered from the centralized procurement information platform of all Chinese provinces. Treatment costs, epidemiological data, and other model parameters were obtained from published literature. The cost of vaccination, treatment costs saved, net costs, cases and deaths averted, life years saved, disability-adjusted life years (DALYs) prevented, and incremental cost-effectiveness ratios were predicted both provincially and nationally. Deterministic sensitivity analyses were used to explore model uncertainty.

**Results:**

The net cost of vaccinating with the domestic bivalent HPV vaccine was the lowest. At the national level, after bivalent or quadrivalent HPV vaccination, the number of cases and deaths averted due to cervical cancer were 12,545 and 5109, respectively, whereas the 9-valent HPV vaccine averted 28,140 cases and 11,459 deaths. HPV vaccines were cost-effective at a national level (maximum cost US$ 18,165 per DALY gained.) compared to the 3 times GDP per capita (US$ 30,837). Bivalent HPV vaccines were cost-effective in all 31 provinces. Imported quadrivalent and 9-valent HPV vaccines were cost-effective in 29 provinces, except Heilongjiang and Gansu. The univariate sensitivity analysis showed that the results were robust when the model parameters were changed, and that the discount rate was the main factor affecting the baseline results.

**Conclusions:**

This study provides evidence that the inclusion of HPV vaccination in the immunization program would be cost-effective at a national level and in most provinces. Provinces with a higher population have more prevented cases, deaths, and DALYs**.** The economics of HPV vaccination at the provincial level differs from that at the national level, and provinces with an inability to pay should seek help from state subsidies.

**Supplementary Information:**

The online version contains supplementary material available at 10.1186/s12889-022-13056-5.

## Background

Human papillomaviruses (HPV) are a large family of epitheliotropic DNA tumor viruses [1]. Approximately 80% of sexually active women are infected with at least one HPV subtype at some point in their lifetimes [2]. Continuous infection with a high-risk HPV subtype is the main cause of cervical cancer [3]. The HPV high-risk subtypes include HPV 16, 18, 31, 33, 35, 39, 45, 51, 52, 56, 58, 59, 66, and 68. Among them, HPV 16 and 18 are highly associated with cervical cancer, and cause about 75% of cervical cancers worldwide [4]. Cervical cancer has become a global public health problem. It is the fourth most common cancer in women. In 2020, an estimated 604,000 women worldwide were diagnosed with cervical cancer, and an estimated 342,000 women died. In China, 110,000 new cases of cervical cancer and 59,000 deaths were reported, therefore having the second-largest burden of cervical cancer in the world [5]. Cervical cancer in China accounts for 18% of the worldwide cervical cancer incidence and 17% of cervical cancer deaths.

As the only cancer that has clear causes, and can be prevented and treated, it is expected to be fully eradicated. Therefore, promoting cervical cancer prevention is of great importance. Vaccination is an effective measure against HPV infection and to reduce cervical cancer incidence. Three prophylactic HPV vaccines are currently available worldwide, including the bivalent, quadrivalent, and 9-valent HPV vaccines [6]. The World Health Organization (WHO) recommends using HPV vaccination as part of routine vaccination in all countries [7]. HPV vaccines are currently used in 129 countries worldwide to prevent HPV-related diseases [8] and have been introduced into the national immunization plans (NIP) of 74 countries [9]. However, the HPV vaccination is not included in China’s NIP.

China has approved HPV vaccines since 2016 [10], including the Cervarix® (GlaxoSmithKline Inc.), Cecolin® (Wantai BioPharm), and Gardasil® and Gardasil® 9 (Merck & Co., Inc.) vaccines. It has a large population and unbalanced economic development among regions. The payment methods for obtaining HPV vaccination services are different among various provinces. Currently, there are three main payment methods for HPV vaccines in China: 1) residents are vaccinated against HPV at their own expense, 2) target populations get free HPV vaccination, such as in Ordos, Inner Mongolia, and Xiamen [11, 12], and 3) medical insurance does a co-payment with residents for the vaccine. However, in Guizhou Province, HPV vaccination is paid through the balance of the employee’s personal medical insurance account [13]. The local government-led free HPV vaccination program is rare in China. Most provinces still need residents to get vaccinated at their own expense, and residents experience a heavy burden of paying for the HPV vaccine. In 2019, China’s Vaccine Management Law authorized provincial governments to increase the types of vaccines available for immunization programs in accordance with the needs of disease prevention and control in their administrative regions [14]. However, China still lacks the economic evidence for HPV vaccine cost-effectiveness at the national and provincial levels. To provide information that could affect policy-making decisions to expand the use of HPV vaccines, we evaluated the economics of all valent HPV vaccines available in the Chinese market at the national and provincial levels.

## Methods

Cost-effectiveness analysis was used to assess the economics of HPV vaccination at a national level and in 31 provinces in Mainland China. From the perspective of the health system, we compared the final cost and health effects of the two strategies of vaccinating and not vaccinating HPV vaccine for women of the target age. The HPV vaccines available in the Chinese market include the domestic bivalent, imported bivalent, imported quadrivalent, and imported 9-valent HPV vaccines. Lifetime effects after vaccination were estimated using the Papillomavirus Rapid Interface for Modeling and Economics (PRIME) model. The results of the economic evaluation were expressed by incremental cost-effectiveness ratios (ICER), and ICER indicators that were constructed based on the disability-adjusted life years (DALYs) were reported. The evaluation assumed that the target population had not been infected with HPV prior to vaccination. Stata15.0 was used to draw a map of China to show the cost-effectiveness results. Sensitivity analysis was conducted to test the impact of six parameters, including target age, discounted rate, vaccine efficacy, procurement, transportation and management cost, and cervical cancer treatment cost, on the robustness of the model results; the results are shown in a tornado diagram. The cost parameters used in the model have been adjusted to 2019 according to the average exchange rate of the RMB against the US dollar and the consumer price index [15]. To eliminate the effect of the time value of money, costs were discounted at a rate of 3%, as recommended by the WHO Guidelines on Health Economics [16]. This study is reported as per the Consolidated Health Economic Evaluation Reporting Standards (CHEERS) guideline (Additional file 1: Table S1) [17].

### PRIME model

The PRIME model was used to conduct an economic evaluation of different HPV vaccines in different provinces and cities in China [18]. The PRIME model is a static proportional outcome model, developed by Jit et al. with support from the WHO [19]. The model aims to aid in the health economic assessment of HPV vaccination for decision-makers and medical workers at all levels. The model considered the association between HPV infection and cervical cancer lesions. Three vaccines are protective against various high-risk HPV subtypes. The bivalent and quadrivalent HPV vaccines are protective against two high-risk HPV subtypes. The 9-valent HPV vaccine is protective against seven high-risk HPV subtypes, which include HPV 16, 18, 31, 33, 45, 52, and 58. We set model parameters based on heterogeneous data from various regions. Table 1 lists the model parameters and their sources.

**Table 1:** PRIME model parameters and data sources

parameter	value	source
Birth cohort size (female)	Additional file	CPSY
Cohort size at vaccination age (female)	Additional file	CPSY
Target age group	9/16	[20]
Vaccine efficacy vs HPV 16/18	100%	[23, 24]
Vaccine efficacy vs HPV 16/18/31/33/45/52/58	100%	[23, 24]
Coverage (all doses)	80%	[26, 25]
Vaccine price per FIG	Additional file	CPP
Vaccine delivery cost per FIG	Additional file	CPP
Total vaccine cost per FIG	Additional file	CPP
Cancer treatment cost (per episode, over lifetime)	US$7547	[22]
DALYs for cancer diagnosis	0.08	[21]
DALYs for non-terminal cancer sequelae (per year)	0.11	[21]
DALYs for terminal cancer	0.78	[21]
Discount rate	3%	[16]
Proportion of cervical cancer cases that are due to HPV 16/18	69.1%	[21]
Proportion of cervical cancer cases that are due to HPV 16/18/31/33/45/52/58	92%	[21]
Discounted GDP per capita	Additional file	CPSY

*CPSY* 20 China and Provincial Statistics Yearbook, *CPP* Centralized procurement platform

### Demographic and regional economic data

The 2019 national and provincial statistics included the number of women born in the cohort and the number of women of target vaccination age in the region. The target age was set according to the age at which HPV vaccination was approved in Mainland China [20]. The target age of vaccination for the bivalent and quadrivalent HPV vaccines was set at 9 years and that for the 9-valent HPV vaccine was set at 16 years.

Regional per capita gross domestic product (GDP) refers to the value of all final products and services produced less the value of products and services used for immediate consumption by all residential units in a region over a period. Since the publication of the Statistical Yearbook data lags behind by 1 year, the demographic and regional economic data used were from the 2020 Statistical Yearbook of each province. Cohort size at vaccination age (female) was calculated from the national population age structure. Data and calculation formulas are shown in Additional file 2: Tables S2–S4 and Additional file 3: Table S5.

### Disease burden data

The disease burden data included epidemiological and economic burden data. Data on the proportion and incidence of and mortality due to cervical cancer caused by different HPV subtypes are obtained from the International Agency for Research on Cancer (IARC) HPV Information Center; data on the DALYs lost due to cervical cancer or death are obtained from the Global Burden of Disease research [21]. The cost of treatment for cervical cancer per capita was based on the cost of treatment for cervical cancer per patient from the time of diagnosis until death [22]. Disease burden data in the model are those for the national level.

### Vaccine efficacy and coverage rates

The vaccine efficacy was based on the proportion of the reduction in the risk of developing cervical cancer associated with the bivalent, quadrivalent and 9-valent HPV vaccines, which we set at 100% [23, 24]. The rollout of HPV vaccination in China is in the initial stages, with coverage data at the national and provincial levels not available. Therefore, the coverage of HPV vaccination was estimated to be 80% [25, 26].

### Vaccination costs

Vaccination costs included per capita vaccine procurement, transportation and management, and service costs. Details of the data are recorded in Additional file 4: Tables S6–S8. The per capita procurement cost was based on the transaction price of the centralized purchase of HPV vaccines by various provinces and cities in China. The per capita vaccine transportation and management, and service costs were based on the transportation fee and service fee of all class II or non-immunization planning vaccines published by various provinces and cities in China. Class II and non-immunization planning vaccines refer to the vaccines received by residents voluntarily, and at their own expense [27].

### Economic evaluation indicators

Costs included the direct and discounted costs, and the incremental costs incurred between receiving HPV vaccines and not receiving them. These costs included vaccination, saved treatment, and net costs. The effect of HPV vaccination was based on the number of cervical cancer cases and deaths averted before and after vaccination. Life-years saved (DALYs averted) were based on the number of life-year (DALY) losses eventually averted due to cervical cancer cases averted by vaccinating a single age cohort in 2019. We also calculated the incremental cost of preventing one case of cervical cancer after HPV vaccination, preventing one death, and of saving the unit DALY. Cost effect is the ratio of the increased cost of saving a unit DALY (cost-effectiveness ratio, CER) and the incremental CER obtained compared to existing standard strategies. ICER of each province was compared with GDP per capita of each region; ICER < 1 times GDP per capita is very cost-effective, 1 < ICER< 3 times per capita GDP is cost-effective, and ICER> 3 times GDP is not cost-effective at all. The calculation formula of the cost and effect index is in Additional file 5.

### Sensitivity analysis

Univariate sensitivity analysis was performed on the target age, vaccine efficacy, procurement cost, transportation and management cost, discount rate, and cervical cancer treatment cost in the model at the national level. The values of three cost-related parameters, including vaccine procurement, transportation and management costs, and cervical cancer treatment cost were adjusted by ±20%; vaccine efficacy was adjusted by − 10% and − 20%; discount rate was adjusted by ±2%; and target age for vaccination was adjusted to 13 and 26 years. We compared the changes in results caused by index changes when the target population was vaccinated with an HPV vaccine, analyzed the robustness of the model, and found the index that had the greatest impact on the results. The uncertainties of the model are summarized by a tornado diagram.

## Results

### Direct cost of HPV vaccines

From the perspective of vaccine valence types, the cost of the domestic bivalent HPV vaccine was the lowest, and the cost of the imported 9-valent HPV vaccine was the highest. The avoidable cost of treatment was highest for the imported 9-valent HPV vaccine, with the cost of treatment being the same for the domestic bivalent, imported bivalent and imported quadrivalent HPV vaccines. This is because the three kinds of vaccines (domestic and imported bivalent and imported quadrivalent) are effective against the same HPV subtypes, and they prevent the same number of cervical cancer cases. In terms of the net cost of prevention and treatment of cervical cancer, the net cost of the domestic bivalent HPV vaccine was the lowest, followed by the imported bivalent, imported quadrivalent and imported 9-valent HPV vaccines. The net cost at the national and provincial levels are shown in Table 2.

**Table 2:** Discounted cost of HPV vaccination for target population at the national and provincial levels (US$)

Province and region	Vaccination costs				Treatment cost savings				Net cost			
	Domestic bivalent vaccine	Imported bivalent vaccine	Imported quadrivalent vaccine	Imported 9-valent vaccine	Domestic bivalent vaccine	Imported bivalent vaccine	Imported quadrivalent vaccine	Imported 9-valent vaccine	Domestic bivalent vaccine	Imported bivalent vaccine	Imported quadrivalent vaccine	Imported 9-valent vaccine
Heilongjiang	22,040,361	38,075,322	52,002,738	115,770,557	2,768,229	2,768,229	2,768,229	5,092,636	19,272,132	35,307,093	49,234,509	110,677,921
Jilin	15,900,744	27,402,289	37,392,137	83,165,994	1,985,593	1,985,593	1,985,593	3,652,832	13,915,151	25,416,696	35,406,543	79,513,162
Liaoning	26,085,902	44,687,292	60,843,820	135,014,094	3,211,290	3,211,290	3,211,290	5,907,720	22,874,612	41,476,002	57,632,530	129,106,374
Hebei	45,768,030	78,219,994	106,406,656	235,901,638	5,602,413	5,602,413	5,602,413	10,306,583	40,165,617	72,617,581	100,804,244	225,595,055
Shanxi	22,481,573	38,422,202	52,267,686	115,876,523	2,751,944	2,751,944	2,751,944	5,062,665	19,729,629	35,670,258	49,515,742	110,813,858
Shandong	60,660,497	104,038,235	141,714,649	314,612,022	7,488,607	7,488,607	7,488,607	13,776,574	53,171,890	96,549,628	134,226,042	300,835,449
Shaanxi	23,037,107	39,605,128	53,995,543	119,983,034	2,860,255	2,860,255	2,860,255	5,261,920	20,176,852	36,744,873	51,135,288	114,721,113
Henan	56,310,467	97,516,746	133,307,105	297,051,359	7,113,732	7,113,732	7,113,732	13,086,924	49,196,735	90,403,014	126,193,373	283,964,435
Anhui	38,920,069	66,131,219	89,765,889	198,553,960	4,697,654	4,697,654	4,697,654	8,642,120	34,222,415	61,433,565	85,068,236	189,911,840
Jiangsu	47,964,187	82,459,477	112,420,902	249,809,742	5,955,167	5,955,167	5,955,167	10,955,541	42,009,021	76,504,311	106,465,735	238,854,202
Hubei	35,932,757	61,267,845	83,273,032	184,445,344	4,373,776	4,373,776	4,373,776	8,046,285	31,558,981	56,894,069	78,899,256	176,399,059
Sichuan	51,203,365	87,002,439	118,096,285	261,218,215	6,180,248	6,180,248	6,180,248	11,369,600	45,023,117	80,822,191	111,916,037	249,848,615
Zhejiang	35,566,628	60,572,516	82,291,772	182,188,048	4,316,944	4,316,944	4,316,944	7,941,749	31,249,684	56,255,572	77,974,829	174,246,299
Hunan	43,503,767	74,791,174	101,966,341	226,578,816	5,401,367	5,401,367	5,401,367	9,936,736	38,102,400	69,389,807	96,564,974	216,642,080
Jiangxi	28,312,597	48,218,382	65,507,863	145,029,669	3,436,477	3,436,477	3,436,477	6,321,980	24,876,120	44,781,905	62,071,387	138,707,689
Yunnan	29,453,709	50,220,618	68,258,043	151,187,989	3,585,139	3,585,139	3,585,139	6,595,458	25,868,570	46,635,479	64,672,904	144,592,530
Guizhou	21,964,296	37,450,649	50,901,564	112,744,316	2,673,519	2,673,519	2,673,519	4,918,383	19,290,777	34,777,130	48,228,045	107,825,933
Fujian	23,613,620	40,596,264	55,346,805	122,985,854	2,931,834	2,931,834	2,931,834	5,393,611	20,681,786	37,664,430	52,414,971	117,592,244
Guangdong	69,454,096	118,700,739	161,474,683	357,987,007	8,501,797	8,501,797	8,501,797	15,640,514	60,952,298	110,198,942	152,972,886	342,346,494
Beijing	12,982,978	22,188,599	30,184,285	66,917,977	1,589,232	1,589,232	1,589,232	2,923,658	11,393,746	20,599,367	28,595,053	63,994,319
Tianjin	9,415,446	16,091,497	21,890,086	48,529,921	1,152,534	1,152,534	1,152,534	2,120,281	8,262,912	14,938,963	20,737,552	46,409,640
Shanghai	14,637,955	25,017,042	34,031,961	75,448,581	1,791,816	1,791,816	1,791,816	3,296,361	12,846,139	23,225,227	32,240,146	72,152,219
Chongqing	18,834,957	32,189,942	43,789,623	97,080,559	2,305,566	2,305,566	2,305,566	4,241,466	16,529,391	29,884,376	41,484,057	92,839,093
Inner Mongolia	15,959,188	26,814,737	36,243,495	79,807,048	1,874,070	1,874,070	1,874,070	3,447,663	14,085,118	24,940,667	34,369,424	76,359,385
Xinjiang	14,909,612	25,694,237	35,061,393	77,981,887	1,861,826	1,861,826	1,861,826	3,425,135	13,047,785	23,832,411	33,199,567	74,556,752
Ningxia	4,105,085	7,074,432	9,653,504	21,470,997	512,619	512,619	512,619	943,053	3,592,466	6,561,812	9,140,885	20,527,943
Tibet	2,071,621	3,570,095	4,871,618	10,835,334	258,692	258,692	258,692	475,912	1,812,929	3,311,403	4,612,926	10,359,422
Guangxi	34,042,388	58,385,772	79,529,600	176,558,372	4,202,571	4,202,571	4,202,571	7,731,330	29,839,817	54,183,201	75,327,029	168,827,042
Qinghai	3,591,889	6,190,024	8,446,674	18,786,893	448,534	448,534	448,534	825,161	3,143,355	5,741,489	7,998,140	17,961,732
Gansu	15,735,046	27,051,509	36,880,601	81,952,195	1,953,641	1,953,641	1,953,641	3,594,058	13,781,405	25,097,868	34,926,959	78,358,138
Hainan	5,615,027	9,653,289	13,160,786	29,244,365	697,154	697,154	697,154	1,282,527	4,917,873	8,956,135	12,463,632	27,961,838
National	840,791,627	1,439,992,866	1,960,438,493	4,312,777,862	103,444,361	103,444,361	103,444,361	188,681,737	737,347,266	1,336,548,506	1,856,994,132	4,124,096,125

### Effect of HPV vaccination on cervical cancer morbidity and mortality

**Fig. 1:**
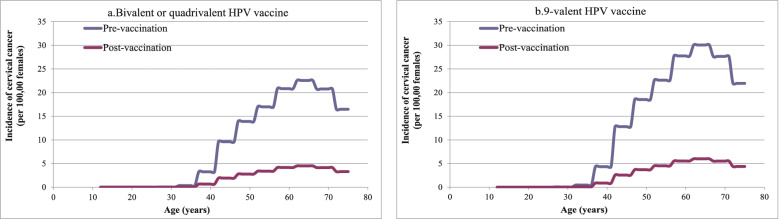
The effect of vaccination on the incidence of cervical cancer by age in 2019

At the national level, the lifetime incidence of cervical cancer for females of all ages, in the same birth cohort, who completed the full courses of the domestic bivalent, imported bivalent, imported quadrivalent, and imported 9-valent HPV vaccines at the target age was significantly lower than that of females in the cohort who did not receive HPV vaccination (Fig. 1). A total of 12,545 cervical cancer cases, and 5109 deaths were averted by the domestic bivalent, imported bivalent and imported quadrivalent HPV vaccines. For the 9-valent HPV vaccine, 28,140 cervical cancer cases and 11,459 deaths were averted.

**Fig. 2:**
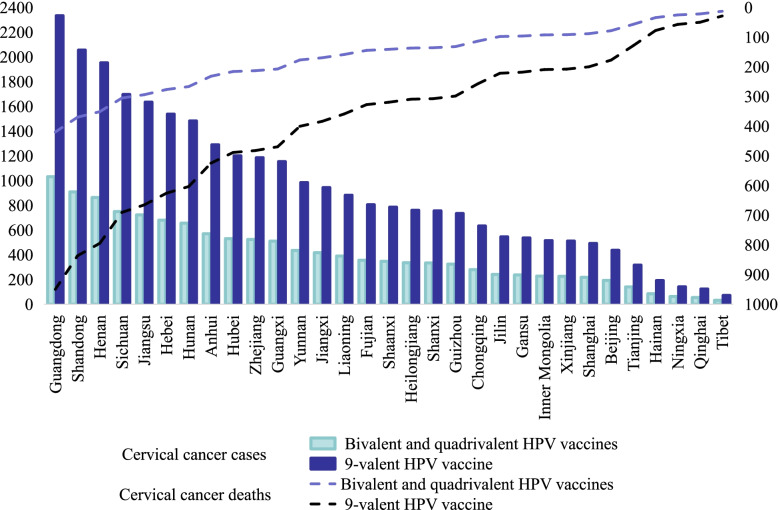
Cervical cancer cases and deaths averted by HPV vaccination at the provincial level in 2019

In 31 provinces, the incidence of cervical cancer decreased with age in the target population after HPV vaccination. The results showed that the number of cervical cancer cases, and deaths averted, life-years saved and DALYs prevented by the imported 9-valent HPV vaccine was higher than the other vaccines. Guangdong province, China’s most populous province, had the most prevented cases, deaths, DALYs, and saved the most life-years, followed by Shandong and Henan provinces. Figure 2 shows the number of cervical cancer cases and deaths adverted by province. The number of cervical cancer cases and deaths averted, DALYs prevented, and life-years saved at a national and provincial level are shown in Additional file 6: Tables S9–S12.

The incremental cost per cervical cancer death averted after HPV vaccination in the target population was much higher than the incremental cost per cervical cancer prevented. The domestic bivalent HPV vaccine was the most cost-effective. This was followed by the imported bivalent, imported 9-valent and imported quadrivalent HPV vaccines. Nationally, the incremental cost of the bivalent HPV vaccine needed to avert cervical cancer cases was US$ 58,779, the incremental cost needed to avert cervical cancer deaths was US$ 144,329, the incremental cost to save one life-year was US$ 7879, and the incremental cost to prevent one DALY was US$ 7213. The incremental costs of cervical cancer cases and deaths averted, saving one life-year, and preventing one DALY with the imported quadrivalent HPV vaccine were US$ 148,034, US$ 363,490, US$ 19,843, and US$ 18,165, respectively (Table 3).

**Table 3:** Incremental costs of various HPV vaccines at the national level (US$)

Vaccines	Incremental cost per			
	Cervical cancer prevented	Life saved	Life year saved	DALY prevented
Domestic bivalent vaccine	58,779	144,329	7879	7213
Imported bivalent vaccine	106,546	261,618	14,282	13,074
Imported quadrivalent vaccine	148,034	363,490	19,843	18,165
Imported 9-valent vaccine	146,554	359,905	18,112	16,939

If the domestic bivalent vaccine with the best performance was used as an intervention for cervical cancer prevention, Heilongjiang province had the lowest incremental cost per case of cervical cancer averted, only requiring US$ 57,410, while Inner Mongolia had the highest incremental cost of US$ 61,977. The minimum cost per cervical cancer death averted in Henan province was US$ 140,032, and the maximum incremental cost per cervical cancer death averted in Inner Mongolia was US$ 152,182. Henan province had the lowest incremental cost per life-year saved, requiring US$ 7644; Inner Mongolia had the highest incremental cost at US$ 8308. When the imported quadrivalent vaccine was used as an intervention to prevent cervical cancer, Henan province had the lowest cost per case of cervical cancer morbidity and mortality, which was US$ 146,284 and US$ 359,193, respectively. Inner Mongolia had the highest incremental costs, with US$ 151,232 for morbidity prevention and US$ 371,343 for mortality prevention. Henan had the lowest incremental cost per life-year saved, at US $19,609, and Inner Mongolia had the highest, at US$ 20,272 (Additional file 7: Tables S13–S16).

### Cost-effectiveness analysis of HPV vaccination

**Fig. 3:**
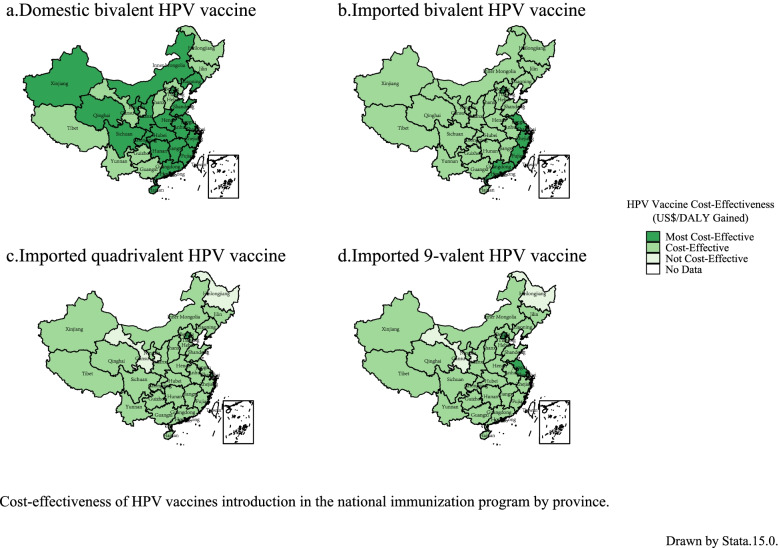
Cost-effectiveness of HPV vaccination in 31 provinces in the target population in 2019

The four HPV vaccines were cost-effective at the national level compared to no HPV vaccination; Among the four vaccines, in 31 provinces, the domestic and imported bivalent HPV vaccines were cost-effective (< 3 times GDP per capita). The domestic bivalent vaccine was very cost-effective (<GDP per capita) in 22 regions including Liaoning, Beijing, Shanghai, Shandong, and Jiangsu. The provinces where the bivalent imported HPV vaccine was most cost-effective were mainly in the eastern coastal cities, including Beijing, Jiangsu, Shanghai, Zhejiang, Fujian, and Guangdong. The imported quadrivalent and imported 9-valent HPV vaccines were cost-effective in 29 provinces. The imported quadrivalent HPV vaccine was very cost-effective in Beijing and Shanghai, and the imported 9-valent HPV vaccine was very cost-effective in Beijing, Shanghai, and Jiangsu. There was no cost-effectiveness for the imported quadrivalent and imported 9-valent HPV vaccines in Heilongjiang and Gansu provinces (>3 times GDP per capita) (Fig. 3). The cost-effectiveness data are shown in Additional file 8: Table S17–S20.

The bivalent and quadrivalent HPV vaccines target the same HPV subtypes, both can protect against HPV 16 and 18, therefore they have the same protective effect against cervical cancer. The 9-valent HPV vaccine had the highest per capita vaccination cost (US$ 574.71), followed by the imported quadrivalent (US$ 357.21), imported bivalent (US$ 262.38), and domestic bivalent HPV vaccines (US$ 153.2). At both the national and provincial levels, the imported bivalent HPV vaccine was approximately 1.7 times the price of the domestic bivalent HPV vaccine, and the imported quadrivalent HPV vaccine was approximately 2.3 times the price of the domestic bivalent vaccine. Therefore, the imported bivalent and imported quadrivalent HPV vaccines were less cost-effective than the domestic bivalent HPV vaccine. The price of the imported quadrivalent HPV vaccine was approximately 1.3 times that of the imported bivalent HPV vaccine, thus making the former less cost-effective. Compared with the bivalent and quadrivalent HPV vaccines, the 9-valent HPV vaccine provides more protection against cervical cancer. Although the price of the 9-valent HPV vaccine is higher, it provides higher protection against HPV. The 9-valent is more cost-effective than the quadrivalent HPV.

### Sensitivity analysis

**Fig. 4:**
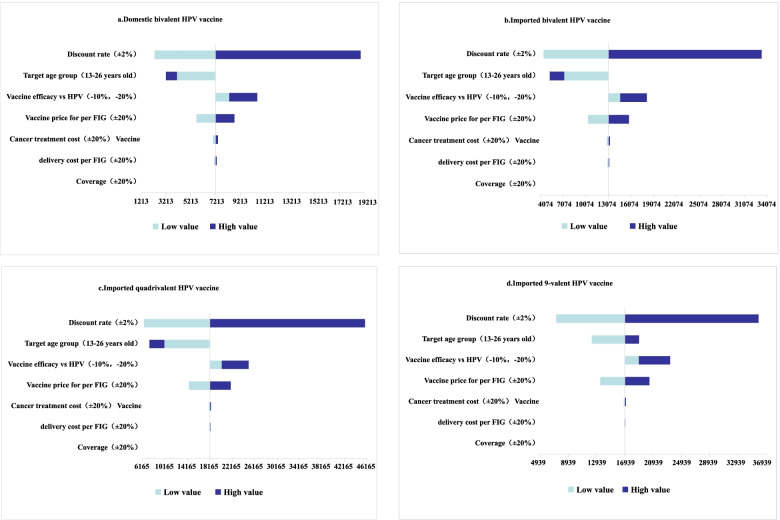
Tornado diagram of the univariate sensitivity analyses (US$/DALY gained)

The model results were robust, and the discount rate was the main factor affecting the baseline results. When the discount rate was adjusted by + 2%, the imported bivalent, imported quadrivalent and imported 9-valent HPV vaccines went from being cost-effective to being cost-ineffective. In the sensitivity analysis of all valent HPV vaccines, the adjustment in discount rate caused the biggest change in the ICER value. After the discount rate was adjusted (±2%), the ICER value ranged from US$ 2311 to US$ 18,959 for the domestic bivalent HPV vaccine. With the imported bivalent HPV vaccine, the ICER value ranged from US$ 4523 to US$ 33,358. ICER values ranged from US$ 6444 to US$ 45,865 after vaccination with the quadrivalent HPV vaccine. ICER values ranged from US$ 7257 to US$ 35,869 after vaccination with the 9-valent HPV vaccine (Fig. 4). The data for the sensitivity analysis are shown in Additional file 9: Table S21.

## Discussion

This study evaluated the cost-effectiveness of HPV vaccination at the national and provincial levels in China compared with no HPV vaccination. Compared with not vaccinating with the HPV vaccine, vaccinating with the HPV vaccine can reduce the incidence of cervical cancer cases in women of all ages. When HPV vaccine coverage reached 80%, for the target population in 2019, introducing bivalent, quadrivalent or 9-valent HPV vaccines into the immunization program could have averted more than 12,545–28,140 cervical cancer cases and approximately 5109–11,459 deaths. Once the HPV vaccine is included in the immunization program, 80% vaccine coverage can be expected. In 2020, Erdos, China, implemented a program for free HPV vaccination for girls aged 13–18 years, and the vaccination rate of the target population reached 85% [28]. The incremental cost of using the domestic bivalent, imported bivalent, imported quadrivalent, and imported 9-valent HPV vaccines for each DALY saved is US$ 7213, US$ 13,074, US$ 18,165, and US$ 16,939, respectively. With 3 times GDP per capita as the threshold, HPV vaccination is cost-effective nationwide. This result is consistent with the research results of HPV vaccination in Vietnam, Australia, South Africa, and other countries [29–31].

With the 3 times per capita GDP as the threshold, the usage of the domestic and imported bivalent HPV vaccines in 31 provinces is cost-effective. Among them, the domestic bivalent vaccine is very cost-effective in 22 of the 31 provinces due to its price advantage. The imported bivalent HPV vaccine is very cost-effective in six economically developed regions (per capita GDP in 2019 > US$ 13,655). Except for Heilongjiang and Gansu, usage of the imported quadrivalent and 9-valent HPV vaccines in other provinces was cost-effective. In the deterministic sensitivity analysis, when the most model parameters were changed, HPV vaccination was still cost-effective.

The net cost of the 9-valent HPV vaccine was higher. However, the 9-valent HPV vaccine protects against more HPV subtypes, prevents more morbidity, and saves more treatment costs than the other vaccines. The reduction in the cost of the 9-valent HPV vaccine can further reduce its net cost. For cervical cancer, bivalent and quadrivalent HPV vaccines have the same protective effect. Currently, the net cost depends on the vaccine price. The bivalent HPV vaccines, especially the domestic bivalent HPV vaccine, have the greatest price advantage and the lowest net cost.

There are provincial differences in the economics of HPV vaccination. The increased cost is either completely worth it, or it is accepted that it is affected by the ICER value and threshold. The ICER values for the domestic bivalent HPV vaccine in Gansu and Beijing were 7138 (US$/DALY gained) and 7254 (US$/DALY gained), respectively; the per capita GDP was US$ 4784 and US$ 23,811, respectively. In Beijing, even if the 9-valent HPV vaccine was used at its highest price, its ICER value was still less than double the per capita GDP. In Gansu, even if the domestically made bivalent HPV vaccine was used at its lowest price, its ICER value was only less than 3 times the per capita GDP. The level of economic development in each province will affect its ability to pay for HPV vaccines. Including the HPV vaccine in the scope of medical insurance payments or state subsidies can increase the availability of HPV vaccines in economically disadvantaged areas.

This study had some limitations. First, our study only considered the protective effect of HPV vaccines on cervical cancer and did not consider the protective effect of HPV vaccines on genital warts, oral cancer, and other diseases, which may have caused the ICER to be overestimated. Second, we assumed that the target age population had not been infected with HPV when entering the model, but there may have been people who were infected with HPV. Third, the impact of cervical cancer screening and HPV transmission on the incidence of cervical cancer was not considered, and the herd immune response was also not considered. Fourth, the vaccine dropout rate, due to side effects after vaccination, was not considered.

## Conclusions

The HPV vaccine being included in the immunization program can reduce the burden of cervical cancer. As a country with a large population, to help accelerate the elimination of cervical cancer, China should include the HPV vaccine in its immunization program as soon as possible. From a provincial perspective, Guangdong, Shandong, Henan, Sichuan, and Jiangsu have benefited from the preventable cervical cancer incidence and avoidable cervical cancer deaths after HPV vaccination, and consideration should be given to including the HPV vaccine in their immunization programs as soon as possible. In various provinces, there is a large gap in the ability to pay for HPV vaccines. To improve the accessibility of HPV vaccines, more attention should be given to economically disadvantaged areas.

## Supplementary Information

**Additional file 1: Table S1.** CHEERS checklist.

**Additional file 2: Table S2.** Cohort size at birth (female) by province; **Table S3f.** Cohort size at vaccination age (9) by province; **Table S4.** Cohort size at vaccination age (16) by province.

**Additional file 3: Table S5.** GDP per capita by province (US$).

**Additional file 4: Table S6.** Provincial Vaccine price per Fig. (US$); **Table S7.** Vaccine delivery cost per FIG by province (US$); **Table S8.** Total vaccine cost per FIG by province (US$).

**Additional file 5.** Calculation formula for cost and effect index.

**Additional file 6: Table S9.** Cervical cancers prevented by province (Case); **Table S10.** Deaths prevented by province (Case); **Table S11.** Life years saved by province; **Table S12.** Nonfatal DALYs prevented by province.

**Additional file 7: Table S13.** Incremental cost per cervical cancer prevented by province (US$); **Table S14.** Incremental cost per life saved by province (US$); **Table S15.** Incremental cost per life year saved by province (US$); **Table S16.** Incremental cost per DALY prevented by province (US$).

**Additional file 8: Table S17.** Cost-effectiveness of the domestic bivalent HPV vaccine; **Table S18.** Cost-effectiveness of the imported bivalent HPV vaccine; **Table S19.** Cost-effectiveness of the quadrivalent HPV vaccine; **Table S20.** Cost-effectiveness of the 9-valent HPV vaccine.

**Additional file 9: Table S21.** Results of sensitivity analyses.

## Data Availability

The datasets generated and analyzed during the current study are available from the additional files.

## Abbreviations

HPV: Human papillomavirus
NIP: National immunization program
WHO: World health organization
ICER: Incremental cost-effectiveness ratios
PRIME: The papillomavirus rapid interface for modelling and economics
GDP: Gross domestic product
CPSY: 2020 China and Provincial Statistics Yearbook
CPP: Centralized procurement platform
DALY: Disability Adjusted of Life Years
