# Supplementary material for: Human papillomavirus vaccination at the national and provincial levels in China: a cost-effectiveness analysis using the PRIME model
Source: BMC Public Health. 2022 Apr 18;22:777. doi: 10.1186/s12889-022-13056-5 (PMC9014632; doi:10.1186/s12889-022-13056-5)
Supplement: Supplementary file 2 — Additional file 2: Table S2. Cohort size at birth (female) by province; Table S3f. Cohort size at vaccination age (9) by province; Table S4. Cohort size at vaccination age (16) by province. [file 12889_2022_13056_MOESM2_ESM.docx]

**Additional file 2.** **Demographic data by province and national**

**Table S2. 2019 Cohort size at birth (female) by province**

| **Province** | **2019 Birth cohort size** |
| --- | --- |
| Heilongjiang | 106,950 |
| Jilin | 81,143 |
| Liaoning | 140,696 |
| Hebei | 406,500 |
| Shanxi | 166,826 |
| Shandong | 585,312 |
| Shaanxi | 197,472 |
| Henan | 593,769 |
| Anhui | 381,147 |
| Jiangsu | 365,666 |
| Hubei | 330,807 |
| Sichuan | 454,420 |
| Zhejiang | 302,615 |
| Hunan | 345,692 |
| Jiangxi | 285,702 |
| Yunnan | 294,336 |
| Guizhou | 240,386 |
| Fujian | 249,390 |
| Guangdong | 689,555 |
| Beijing | 860,70 |
| Tianjin | 48,030 |
| Shanghai | 46,193 |
| Chongqing | 152,460 |
| Inner Mongolia | 101,323 |
| Xinjiang | 101,803 |
| Ningxia | 47,040 |
| Tibet | 25,662 |
| Guangxi | 312,558 |
| Qinghai | 40,282 |
| Gansu | 137,494 |
| Hainan | 58,246 |
| National | 7,176,331 |

**Table S3. Cohort size at vaccination age(9) by province**

| **Province** | **Target age (9) group** |
| --- | --- |
| Heilongjiang | 183,584 |
| Jilin | 131,681 |
| Liaoning | 212,967 |
| Hebei | 371,542 |
| Shanxi | 182,504 |
| Shandong | 496,631 |
| Shaanxi | 189,687 |
| Henan | 471,770 |
| Anhui | 311,540 |
| Jiangsu | 394,936 |
| Hubei | 290,061 |
| Sichuan | 409,863 |
| Zhejiang | 286,292 |
| Hunan | 358,209 |
| Jiangxi | 227,901 |
| Yunnan | 237,760 |
| Guizhou | 177,303 |
| Fujian | 194,434 |
| Guangdong | 563,824 |
| Beijing | 105,395 |
| Tianjin | 76,434 |
| Shanghai | 118,830 |
| Chongqing | 152,901 |
| Inner Mongolia | 124,285 |
| Xinjiang | 123,473 |
| Ningxia | 33,996 |
| Tibet | 17,156 |
| Guangxi | 278,707 |
| Qinghai | 29,746 |
| Gansu | 129,562 |
| Hainan | 46,234 |
| National | 6,860,245 |

**Table S4. Cohort size at vaccination age(16) by province**

| **Province** | **Target age (16) group** |
| --- | --- |
| Heilongjiang | 253,181 |
| Jilin | 181,601 |
| Liaoning | 293,703 |
| Hebei | 512,393 |
| Shanxi | 251,691 |
| Shandong | 684,904 |
| Shaanxi | 261,597 |
| Henan | 650,618 |
| Anhui | 429,644 |
| Jiangsu | 544,656 |
| Hubei | 400,022 |
| Sichuan | 565,241 |
| Zhejiang | 394,825 |
| Hunan | 494,006 |
| Jiangxi | 314,298 |
| Yunnan | 327,894 |
| Guizhou | 244,518 |
| Fujian | 268,144 |
| Guangdong | 777,570 |
| Beijing | 145,350 |
| Tianjin | 105,410 |
| Shanghai | 163,879 |
| Chongqing | 210,865 |
| Inner Mongolia | 171,401 |
| Xinjiang | 170,281 |
| Ningxia | 46,884 |
| Tibet | 23,660 |
| Guangxi | 384,364 |
| Qinghai | 41,023 |
| Gansu | 178,679 |
| Hainan | 63,761 |
| National | 9,380,335 |

Number of Target Age Population=(National Number of Target Age Women/National Population in 2019) ^1^ * Total number of National (provincial) Statistical Yearbook Population in 2019

**References**

1 Internal data are from China 2010 census data.
