# Supplementary material for: Human papillomavirus vaccination at the national and provincial levels in China: a cost-effectiveness analysis using the PRIME model
Source: BMC Public Health. 2022 Apr 18;22:777. doi: 10.1186/s12889-022-13056-5 (PMC9014632; doi:10.1186/s12889-022-13056-5)
Supplement: Supplementary file 3 — Additional file 3: Table S5. GDP per capita by province (US$). [file 12889_2022_13056_MOESM3_ESM.docx]

**Additional file 3.** **2019 Discounted Data for GDP per capita by province (US$)**

**Table S5. 2019 Discounted GDP per capita by province (US$)**

| **Province** | **2019 GDP per capita** |
| --- | --- |
| Heilongjiang | 5,246 |
| Jilin | 6,304 |
| Liaoning | 8,293 |
| Hebei | 6,720 |
| Shanxi | 6,630 |
| Shandong | 10,244 |
| Shaanxi | 9,664 |
| Henan | 8,176 |
| Anhui | 8,482 |
| Jiangsu | 17,923 |
| Hubei | 11,221 |
| Sichuan | 8,087 |
| Zhejiang | 15,605 |
| Hunan | 8,343 |
| Jiangxi | 7,709 |
| Yunnan | 6,952 |
| Guizhou | 6,733 |
| Fujian | 15,535 |
| Guangdong | 13,655 |
| Beijing | 23,811 |
| Tianjin | 13,094 |
| Shanghai | 22,805 |
| Chongqing | 10,995 |
| Inner Mongolia | 9,838 |
| Xinjiang | 7,870 |
| Ningxia | 7,861 |
| Tibet | 7,091 |
| Guangxi | 6,230 |
| Qinghai | 7,102 |
| Gansu | 4,784 |
| Hainan | 8,193 |
| National | 10,279 |
