# Supplementary material for: Human papillomavirus vaccination at the national and provincial levels in China: a cost-effectiveness analysis using the PRIME model
Source: BMC Public Health. 2022 Apr 18;22:777. doi: 10.1186/s12889-022-13056-5 (PMC9014632; doi:10.1186/s12889-022-13056-5)
Supplement: Supplementary file 4 — Additional file 4: Table S6. Provincial Vaccine price per Fig. (US$); Table S7. Vaccine delivery cost per FIG by province (US$); Table S8. Total vaccine cost per FIG by province (US$). [file 12889_2022_13056_MOESM4_ESM.docx]

**Additional file 4.** **Vaccine discounted cost per FIG by province (US$)**

The procurement costs is the transaction price of the centralized purchase of HPV vaccine by various provinces and cities in China. National and provincial prices of HPV vaccines are consistent. The all-dose prices of centralized procurement in 2019 are shown in Table S5:

**Table S6. Provincial Vaccine discounted price per FIG (US$)**

| **Domestic bivalent HPV vaccine** | **Imported bivalent HPV vaccine** | **Quadrivalent HPV vaccine** | **9-valent HPV vaccine** |
| --- | --- | --- | --- |
| 143.11 | 252.29 | 347.12 | 564.62 |

FIG:Fully vaccinated girl

**Table S7.** **Vaccine delivery discounted cost per FIG by province (US$)**

| **Province** | **Vaccine delivery cost per FIG** |
| --- | --- |
|  |  |
| Heilongjiang | 6.96 |
| Jilin | 7.83 |
| Liaoning | 10 |
| Hebei | 10.87 |
| Shanxi | 10.87 |
| Shandong | 9.57 |
| Shaanxi | 8.7 |
| Henan | 6.09 |
| Anhui | 13.05 |
| Jiangsu | 8.7 |
| Hubei | 11.74 |
| Sichuan | 13.05 |
| Zhejiang | 12.18 |
| Hunan | 8.7 |
| Jiangxi | 12.18 |
| Yunnan | 11.74 |
| Guizhou | 11.74 |
| Fujian | 8.7 |
| Guangdong | 10.87 |
| Beijing | 10.87 |
| Tianjin | 10.87 |
| Shanghai | 10.87 |
| Chongqing | 10.87 |
| Inner Mongolia | 17.4 |
| Xinjiang | 7.83 |
| Ningxia | 7.83 |
| Tibet | 7.83 |
| Guangxi | 9.57 |
| Qinghai | 7.83 |
| Gansu | 8.7 |
| Hainan | 8.7 |
| National | 10.09 |

**Table S8. Total Vaccine discounted cost per FIG by province (US$)**

|  | **Total Vaccine cost per FIG** | | | |
| --- | --- | --- | --- | --- |
| **Province** | **Domestic bivalent**  **HPV vaccine** | **Imported bivalent**  **HPV vaccine** | **Quadrivalent HPV vaccine** | **9-valent HPV vaccine** |
| Heilongjiang | 150.07 | 259.25 | 354.08 | 571.58 |
| Jilin | 150.94 | 260.12 | 354.95 | 572.45 |
| Liaoning | 153.11 | 262.29 | 357.12 | 574.62 |
| Hebei | 153.98 | 263.16 | 357.99 | 575.49 |
| Shanxi | 153.98 | 263.16 | 357.99 | 575.49 |
| Shandong | 152.68 | 261.86 | 356.69 | 574.19 |
| Shaanxi | 151.81 | 260.99 | 355.82 | 573.32 |
| Henan | 149.2 | 258.38 | 353.21 | 570.71 |
| Anhui | 156.16 | 265.34 | 360.17 | 577.67 |
| Jiangsu | 151.81 | 260.99 | 355.82 | 573.32 |
| Hubei | 154.85 | 264.03 | 358.86 | 576.36 |
| Sichuan | 156.16 | 265.34 | 360.17 | 577.67 |
| Zhejiang | 155.29 | 264.47 | 359.3 | 576.8 |
| Hunan | 151.81 | 260.99 | 355.82 | 573.32 |
| Jiangxi | 155.29 | 264.47 | 359.3 | 576.8 |
| Yunnan | 154.85 | 264.03 | 358.86 | 576.36 |
| Guizhou | 154.85 | 264.03 | 358.86 | 576.36 |
| Fujian | 151.81 | 260.99 | 355.82 | 573.32 |
| Guangdong | 153.98 | 263.16 | 357.99 | 575.49 |
| Beijing | 153.98 | 263.16 | 357.99 | 575.49 |
| Tianjin | 153.98 | 263.16 | 357.99 | 575.49 |
| Shanghai | 153.98 | 263.16 | 357.99 | 575.49 |
| Chongqing | 153.98 | 263.16 | 357.99 | 575.49 |
| Inner Mongolia | 160.51 | 269.69 | 364.52 | 582.02 |
| Xinjiang | 150.94 | 260.12 | 354.95 | 572.45 |
| Ningxia | 150.94 | 260.12 | 354.95 | 572.45 |
| Tibet | 150.94 | 260.12 | 354.95 | 572.45 |
| Guangxi | 152.68 | 261.86 | 356.69 | 574.19 |
| Qinghai | 150.94 | 260.12 | 354.95 | 572.45 |
| Gansu | 151.81 | 260.99 | 355.82 | 573.32 |
| Hainan | 151.81 | 260.99 | 355.82 | 573.32 |
| National | 153.2 | 262.38 | 357.21 | 574.71 |

Quadrivalent HPV vaccine: Imported Quadrivalent HPV vaccine; 9-valent HPV vaccine : Imported 9-valent HPV vaccine
