# Supplementary material for: Human papillomavirus vaccination at the national and provincial levels in China: a cost-effectiveness analysis using the PRIME model
Source: BMC Public Health. 2022 Apr 18;22:777. doi: 10.1186/s12889-022-13056-5 (PMC9014632; doi:10.1186/s12889-022-13056-5)
Supplement: Supplementary file 5 — Additional file 5. Calculation formula for cost and effect index. [file 12889_2022_13056_MOESM5_ESM.docx]

**Additional file 5. Calculation formula of cost and effect indexs**

1. **Cost**
2. **Vaccination cost**

The total cost of vaccinating a single age cohort in the base year.

=Total cost of vaccine for a single female with full vaccination×number of female of target age×vaccine coverage

1. **The cost of treatment saved**

The treatment costs eventually averted due to cervical cancer cases prevented by vaccinating a single age cohort in the base year.

=Number of cervical cancer cases prevented×Cost of cervical cancer treatment

1. **Net cost**

**The net cost of vaccinating a single age cohort in the base year. This is equal to the cost of vaccination minus the treatment costs saved.**

=Vaccination cost-The cost of treatment saved

1. **Cost-effectiveness**
2. **Number of cervical cancer cases prevented**

=(Incidence rate of vaccine-specific cervical cancer among unvaccinated in the target age population in the future-Incidence rate of vaccine-specific cervical cancer among vaccinated in the target age population in the future)×Number of women vaccinated at the target age

1. **Number of cervical cancer deaths prevented**

=(Mortality of vaccine-specific cervical cancer among unvaccinated in the target age population in the future-Mortality of vaccine-specific cervical cancer among vaccinated in the target age population in the future)×Number of women vaccinated at the target age

1. **Life years saved**

=Number of cervical cancer deaths prevented×remaining life years

1. **Incremental cost of per case** **cervical cancer prevented**

=Net cost/Number of cervical cancer prevented

1. **Incremental cost of per** **deaths prevented**

=Net cost/Number of deaths prevented

1. **Incremental cost of per life years saved**

=Net cost/Life year saved

Cost effect is the ratio of the increased cost of saving unit DALY(cost‑effectiveness ratio, CER),and the incremental cost-effectiveness ratio obtained compared to existing standard strategies.CER can be used instead of ICER for calculation when compared with no intervention.^1^In our study, the results after vaccination were compared with those without vaccination, so CER was used instead of ICER for calculation. ICER of each province was compared with GDP per capita of each region, ICER <1 times GDP per capita is very cost effectiveness,1<ICER<3 times per capita GDP has cost effect, there is no cost-effectiveness at ICER>3 times GDP.^2^

1. **Incremental cost per DALY prevented(ICER)**

=Net cost/(Life years saved+Nonfatal DALYs averted)

1. Life years saved=Deaths prevented×Remaining life years
2. Nonfatal DALYs averted=[Cervical cancers prevented-Deaths prevented]×DALYs for Nonfatal cancer+Deaths prevented×DALYs for Fatal cancer
3. DALYs for Nonfatal cancer=DALYs for cancer diagnosis+DALYs for non-terminal cancer sequelae (per year)×4
4. DALYs for Fatal cancer=DALYs for cancer diagnosis+DALYs for terminal cancer

Death probability: Age specific death-rate;

Survival probability:1-Death probability;

Number of survivors: Set 0 years old as 1, the number of survivors in the previous age group×survival probability;

Survival person-year: The mean number of standardized survivors in adjacent age groups;

Total survival person-year: sum of survival person-year;

Residual life: Residual life=Total survival person-year/number of survivors;

Discounted residual life: Discounted residual life=The cumulative discount factor corresponding to undiscounted residual life+Undiscounted residual life of less than one year

**References**

1. Bi Zhaofeng, Li Yafei, Wei Feixue, et al.Introduction of rapid interface model of HPV vaccine modeling and economic evaluation and example analysis ofChina[J].The Chinese Journal of Preventive Medicine,2019, 53(7):744-751.[In chinese]

2. Baltussen R, Taghreed A, Torres T T, et al.Making Choices in Health:WHO Guide to Cost-Effectiveness Analysis.World Health Organization, 20
