# Supplementary material for: Human papillomavirus vaccination at the national and provincial levels in China: a cost-effectiveness analysis using the PRIME model
Source: BMC Public Health. 2022 Apr 18;22:777. doi: 10.1186/s12889-022-13056-5 (PMC9014632; doi:10.1186/s12889-022-13056-5)
Supplement: Supplementary file 6 — Additional file 6: Table S9. Cervical cancers prevented by province (Case); Table S10. Deaths prevented by province (Case); Table S11. Life years saved by province; Table S12. Nonfatal DALYs prevented by province. [file 12889_2022_13056_MOESM6_ESM.docx]

**Additional file 6. Cervical cancers cases prevented and deaths averted by province**

**Table S9.** **Cervical cancers prevented by province (Case)**

| **Province** | **Cervical cancers prevented** **(Case)** | | | |
| --- | --- | --- | --- | --- |
|  | **Domestic bivalent HPV vaccine** | **Imported bivalent HPV vaccine** | **Quadrivalent HPV vaccine** | **9-valent HPV vaccine** |
| Heilongjiang | 336 | 336 | 336 | 760 |
| Jilin | 241 | 241 | 241 | 545 |
| Liaoning | 389 | 389 | 389 | 881 |
| Hebei | 679 | 679 | 679 | 1,537 |
| Shanxi | 334 | 334 | 334 | 755 |
| Shandong | 908 | 908 | 908 | 2,055 |
| Shaanxi | 347 | 347 | 347 | 785 |
| Henan | 863 | 863 | 863 | 1,952 |
| Anhui | 570 | 570 | 570 | 1,289 |
| Jiangsu | 722 | 722 | 722 | 1,634 |
| Hubei | 530 | 530 | 530 | 1,200 |
| Sichuan | 749 | 749 | 749 | 1,696 |
| Zhejiang | 524 | 524 | 524 | 1,184 |
| Hunan | 655 | 655 | 655 | 1,482 |
| Jiangxi | 417 | 417 | 417 | 943 |
| Yunnan | 435 | 435 | 435 | 984 |
| Guizhou | 324 | 324 | 324 | 734 |
| Fujian | 356 | 356 | 356 | 804 |
| Guangdong | 1,031 | 1,031 | 1,031 | 2,333 |
| Beijing | 193 | 193 | 193 | 436 |
| Tianjin | 140 | 140 | 140 | 316 |
| Shanghai | 217 | 217 | 217 | 492 |
| Chongqing | 280 | 280 | 280 | 633 |
| Inner Mongolia | 227 | 227 | 227 | 514 |
| Xinjiang | 226 | 226 | 226 | 511 |
| Ningxia | 62 | 62 | 62 | 141 |
| Tibet | 31 | 31 | 31 | 71 |
| Guangxi | 510 | 510 | 510 | 1,153 |
| Qinghai | 54 | 54 | 54 | 123 |
| Gansu | 237 | 237 | 237 | 536 |
| Hainan | 85 | 85 | 85 | 191 |
| National | 12,545 | 12,545 | 12,545 | 28,140 |

**Table S10.** **Deaths prevented by province (Case)**

| **Province** | **Deaths prevented (Case)** | | | |
| --- | --- | --- | --- | --- |
|  | **Imported bivalent HPV vaccine** | **Domestic bivalent HPV vaccine** | **Quadrivalent HPV vaccine** | **9-valent HPV vaccine** |
| Heilongjiang | 137 | 137 | 137 | 309 |
| Jilin | 98 | 98 | 98 | 222 |
| Liaoning | 159 | 159 | 159 | 359 |
| Hebei | 277 | 277 | 277 | 626 |
| Shanxi | 136 | 136 | 136 | 307 |
| Shandong | 370 | 370 | 370 | 837 |
| Shaanxi | 141 | 141 | 141 | 320 |
| Henan | 351 | 351 | 351 | 795 |
| Anhui | 232 | 232 | 232 | 525 |
| Jiangsu | 294 | 294 | 294 | 665 |
| Hubei | 216 | 216 | 216 | 489 |
| Sichuan | 305 | 305 | 305 | 690 |
| Zhejiang | 213 | 213 | 213 | 482 |
| Hunan | 267 | 267 | 267 | 603 |
| Jiangxi | 170 | 170 | 170 | 384 |
| Yunnan | 177 | 177 | 177 | 401 |
| Guizhou | 132 | 132 | 132 | 299 |
| Fujian | 145 | 145 | 145 | 328 |
| Guangdong | 420 | 420 | 420 | 950 |
| Beijing | 78 | 78 | 78 | 178 |
| Tianjin | 57 | 57 | 57 | 129 |
| Shanghai | 88 | 88 | 88 | 200 |
| Chongqing | 114 | 114 | 114 | 258 |
| Inner Mongolia | 93 | 93 | 93 | 209 |
| Sinkiang | 92 | 92 | 92 | 208 |
| Ningxia | 25 | 25 | 25 | 57 |
| Tibet | 13 | 13 | 13 | 29 |
| Guangxi | 208 | 208 | 208 | 470 |
| Qinghai | 22 | 22 | 22 | 50 |
| Gansu | 96 | 96 | 96 | 218 |
| Hainan | 34 | 34 | 34 | 78 |
| National | 5,109 | 5,109 | 5,109 | 11,459 |

**Table S11.** **Life years saved by province**

| **Province** | **Life years saved** | |
| --- | --- | --- |
|  | **Bivalent and Quadrivalent HPV vaccine** | **9-valent HPV vaccine** |
| Heilongjiang | 2,504 | 6,146 |
| Jilin | 1,796 | 4,408 |
| Liaoning | 2,905 | 7,129 |
| Hebei | 5,068 | 12,438 |
| Shanxi | 2,490 | 6,110 |
| Shandong | 6,775 | 16,626 |
| Shaanxi | 2,588 | 6,350 |
| Henan | 6,436 | 15,793 |
| Anhui | 4,250 | 10,429 |
| Jiangsu | 5,388 | 13,221 |
| Hubei | 3,957 | 9,710 |
| Sichuan | 5,591 | 13,721 |
| Zhejiang | 3,905 | 9,584 |
| Hunan | 4,886 | 11,992 |
| Jiangxi | 3,109 | 7,629 |
| Yunnan | 3,243 | 7,959 |
| Guizhou | 2,419 | 5,936 |
| Fujian | 2,652 | 6,509 |
| Guangdong | 7,691 | 18,875 |
| Beijing | 1,438 | 3,528 |
| Tianjin | 1,043 | 2,559 |
| Shanghai | 1,621 | 3,978 |
| Chongqing | 2,086 | 5,119 |
| Inner Mongolia | 1,695 | 4,161 |
| Sinkiang | 1,684 | 4,133 |
| Ningxia | 464 | 1,138 |
| Tibet | 234 | 574 |
| Guangxi | 3,802 | 9,330 |
| Qinghai | 406 | 996 |
| Gansu | 1,767 | 4,337 |
| Hainan | 631 | 1,548 |
| National | 93,584 | 227,702 |

**Table S12.** **Nonfatal DALYs prevented by province**

| **Province** | **Nonfatal DALYs prevented** | |
| --- | --- | --- |
|  | **Bivalent and Quadrivalent HPV vaccine** | **9-valent HPV vaccine** |
| Heilongjiang | 231 | 426 |
| Jilin | 166 | 305 |
| Liaoning | 268 | 494 |
| Hebei | 468 | 861 |
| Shanxi | 230 | 423 |
| Shandong | 626 | 1,151 |
| Shaanxi | 239 | 440 |
| Henan | 595 | 1,094 |
| Anhui | 393 | 722 |
| Jiangsu | 498 | 916 |
| Hubei | 366 | 673 |
| Sichuan | 517 | 950 |
| Zhejiang | 361 | 664 |
| Hunan | 451 | 831 |
| Jiangxi | 287 | 528 |
| Yunnan | 300 | 551 |
| Guizhou | 223 | 411 |
| Fujian | 245 | 451 |
| Guangdong | 711 | 1,307 |
| Beijing | 133 | 244 |
| Tianjin | 96 | 177 |
| Shanghai | 150 | 276 |
| Chongqing | 193 | 355 |
| Inner Mongolia | 157 | 288 |
| Sinkiang | 156 | 286 |
| Ningxia | 43 | 79 |
| Tibet | 22 | 40 |
| Guangxi | 351 | 646 |
| Qinghai | 37 | 69 |
| Gansu | 163 | 300 |
| Hainan | 58 | 107 |
| National | 8,646 | 15,770 |
