# Supplementary material for: Human papillomavirus vaccination at the national and provincial levels in China: a cost-effectiveness analysis using the PRIME model
Source: BMC Public Health. 2022 Apr 18;22:777. doi: 10.1186/s12889-022-13056-5 (PMC9014632; doi:10.1186/s12889-022-13056-5)
Supplement: Supplementary file 7 — Additional file 7: Table S13. Incremental cost per cervical cancer prevented by province (US$); Table S14. Incremental cost per life saved by province (US$); Table S15. Incremental cost per life year saved by province (US$); Table S16. Incremental cost per DALY prevented by province (US$). [file 12889_2022_13056_MOESM7_ESM.docx]

**Additional file 7.** **Data for various incremental cost**

**Table S13.** **Incremental cost per cervical cancer prevented by province(US$)**

| **Index** | **Incremental cost per cervical cancer prevented** | | | |
| --- | --- | --- | --- | --- |
| **Province** | **Domestic bivalent HPV vaccine** | **Imported bivalent HPV vaccine** | **Quadrivalent HPV vaccine** | **9-valent HPV vaccine** |
| Heilongjiang | 57,410 | 105,176 | 146,665 | 145,719 |
| Jilin | 57,790 | 105,557 | 147,046 | 145,951 |
| Liaoning | 58,740 | 106,506 | 147,995 | 146,530 |
| Hebei | 59,120 | 106,887 | 148,376 | 146,762 |
| Shanxi | 59,120 | 106,887 | 148,376 | 146,762 |
| Shandong | 58,552 | 106,318 | 147,807 | 146,415 |
| Shaanxi | 58,171 | 105,938 | 147,426 | 146,183 |
| Henan | 57,029 | 104,796 | 146,284 | 145,487 |
| Anhui | 60,074 | 107,841 | 149,329 | 147,343 |
| Jiangsu | 58,171 | 105,938 | 147,426 | 146,183 |
| Hubei | 59,501 | 107,268 | 148,756 | 146,994 |
| Sichuan | 60,074 | 107,841 | 149,329 | 147,343 |
| Zhejiang | 59,694 | 107,460 | 148,949 | 147,111 |
| Hunan | 58,171 | 105,938 | 147,426 | 146,183 |
| Jiangxi | 59,694 | 107,460 | 148,949 | 147,111 |
| Yunnan | 59,501 | 107,268 | 148,756 | 146,994 |
| Guizhou | 59,501 | 107,268 | 148,756 | 146,994 |
| Fujian | 58,171 | 105,938 | 147,426 | 146,183 |
| Guangdong | 59,120 | 106,887 | 148,376 | 146,762 |
| Beijing | 59,120 | 106,887 | 148,376 | 146,762 |
| Tianjin | 59,120 | 106,887 | 148,376 | 146,762 |
| Shanghai | 59,120 | 106,887 | 148,376 | 146,762 |
| Chongqing | 59,120 | 106,887 | 148,376 | 146,762 |
| Inner Mongolia | 61,977 | 109,744 | 151,232 | 148,503 |
| Xinjiang | 57,790 | 105,557 | 147,046 | 145,951 |
| Ningxia | 57,790 | 105,557 | 147,046 | 145,951 |
| Tibet | 57,790 | 105,557 | 147,046 | 145,951 |
| Guangxi | 58,552 | 106,318 | 147,807 | 146,415 |
| Qinghai | 57,790 | 105,557 | 147,046 | 145,951 |
| Gansu | 58,171 | 105,938 | 147,426 | 146,183 |
| Hainan | 58,171 | 105,938 | 147,426 | 146,183 |
| National | 58,779 | 106,546 | 148,034 | 146,554 |

**Table S14.** **Incremental cost per life saved by province(US$)**

| **Index** | **Incremental cost per life saved** | | | |
| --- | --- | --- | --- | --- |
| **Province** | **Domestic bivalent HPV vaccine** | **Imported bivalent HPV vaccine** | **Quadrivalent HPV vaccine** | **9-valent HPV vaccine** |
| Heilongjiang | 140,967 | 258,255 | 360,128 | 357,855 |
| Jilin | 141,901 | 259,190 | 361,062 | 358,425 |
| Liaoning | 144,233 | 261,521 | 363,394 | 359,846 |
| Hebei | 145,167 | 262,456 | 364,328 | 360,416 |
| Shanxi | 145,167 | 262,456 | 364,328 | 360,416 |
| Shandong | 143,771 | 261,059 | 362,932 | 359,565 |
| Shaanxi | 142,836 | 260,124 | 361,997 | 358,995 |
| Henan | 140,032 | 257,321 | 359,193 | 357,286 |
| Anhui | 147,509 | 264,797 | 366,670 | 361,844 |
| Jiangsu | 142,836 | 260,124 | 361,997 | 358,995 |
| Hubei | 146,102 | 263,390 | 365,263 | 360,986 |
| Sichuan | 147,509 | 264,797 | 366,670 | 361,844 |
| Zhejiang | 146,574 | 263,863 | 365,735 | 361,274 |
| Hunan | 142,836 | 260,124 | 361,997 | 358,995 |
| Jiangxi | 146,574 | 263,863 | 365,735 | 361,274 |
| Yunnan | 146,102 | 263,390 | 365,263 | 360,986 |
| Guizhou | 146,102 | 263,390 | 365,263 | 360,986 |
| Fujian | 142,836 | 260,124 | 361,997 | 358,995 |
| Guangdong | 145,167 | 262,456 | 364,328 | 360,416 |
| Beijing | 145,167 | 262,456 | 364,328 | 360,416 |
| Tianjin | 145,167 | 262,456 | 364,328 | 360,416 |
| Shanghai | 145,167 | 262,456 | 364,328 | 360,416 |
| Chongqing | 145,167 | 262,456 | 364,328 | 360,416 |
| Inner Mongolia | 152,182 | 269,470 | 371,343 | 364,692 |
| Xinjiang | 141,901 | 259,190 | 361,062 | 358,425 |
| Ningxia | 141,901 | 259,190 | 361,062 | 358,425 |
| Tibet | 141,901 | 259,190 | 361,062 | 358,425 |
| Guangxi | 143,771 | 261,059 | 362,932 | 359,565 |
| Qinghai | 141,901 | 259,190 | 361,062 | 358,425 |
| Gansu | 142,836 | 260,124 | 361,997 | 358,995 |
| Hainan | 142,836 | 260,124 | 361,997 | 358,995 |
| National | 144,329 | 261,618 | 363,490 | 359,905 |

**Table S15. Incremental cost per life year saved by province(US$)**

| **Index** | **Incremental cost per life year saved** | | | |
| --- | --- | --- | --- | --- |
| **Province** | **Domestic bivalent HPV vaccine** | **Imported bivalent HPV vaccine** | **Quadrivalent H**PV vaccine | **9-valent H**PV vaccine |
| Heilongjiang | 7,695 | 14,098 | 19,660 | 18,009 |
| Jilin | 7,746 | 14,149 | 19,711 | 18,037 |
| Liaoning | 7,874 | 14,277 | 19,838 | 18,109 |
| Hebei | 7,925 | 14,328 | 19,889 | 18,138 |
| Shanxi | 7,925 | 14,328 | 19,889 | 18,138 |
| Shandong | 7,849 | 14,251 | 19,813 | 18,095 |
| Shaanxi | 7,797 | 14,200 | 19,762 | 18,066 |
| Henan | 7,644 | 14,047 | 19,609 | 17,980 |
| Anhui | 8,053 | 14,455 | 20,017 | 18,209 |
| Jiangsu | 7,797 | 14,200 | 19,762 | 18,066 |
| Hubei | 7,976 | 14,379 | 19,940 | 18,166 |
| Sichuan | 8,053 | 14,455 | 20,017 | 18,209 |
| Zhejiang | 8,002 | 14,404 | 19,966 | 18,181 |
| Hunan | 7,797 | 14,200 | 19,762 | 18,066 |
| Jiangxi | 8,002 | 14,404 | 19,966 | 18,181 |
| Yunnan | 7,976 | 14,379 | 19,940 | 18,166 |
| Guizhou | 7,976 | 14,379 | 19,940 | 18,166 |
| Fujian | 7,797 | 14,200 | 19,762 | 18,066 |
| Guangdong | 7,925 | 14,328 | 19,889 | 18,138 |
| Beijing | 7,925 | 14,328 | 19,889 | 18,138 |
| Tianjin | 7,925 | 14,328 | 19,889 | 18,138 |
| Shanghai | 7,925 | 14,328 | 19,889 | 18,138 |
| Chongqing | 7,925 | 14,328 | 19,889 | 18,138 |
| Inner Mongolia | 8,308 | 14,711 | 20,272 | 18,353 |
| Xinjiang | 7,746 | 14,149 | 19,711 | 18,037 |
| Ningxia | 7,746 | 14,149 | 19,711 | 18,037 |
| Tibet | 7,746 | 14,149 | 19,711 | 18,037 |
| Guangxi | 7,849 | 14,251 | 19,813 | 18,095 |
| Qinghai | 7,746 | 14,149 | 19,711 | 18,037 |
| Gansu | 7,797 | 14,200 | 19,762 | 18,066 |
| Hainan | 7,797 | 14,200 | 19,762 | 18,066 |
| National | 7,879 | 14,282 | 19,843 | 18,112 |

**Table S16. Incremental cost per DALY prevented by province(US$)**

| **Index** | **Incremental cost per DALY prevented (ICER)** | | | |
| --- | --- | --- | --- | --- |
| **Province** | **Domestic bivalent HPV vaccine** | **Imported bivalent HPV vaccine** | **Quadrivalent HPV vaccine** | **9-valent HPV vaccine** |
| Heilongjiang | 7,045 | 12,906 | 17,997 | 16,842 |
| Jilin | 7,091 | 12,953 | 18,044 | 16,869 |
| Liaoning | 7,208 | 13,069 | 18,160 | 16,936 |
| Hebei | 7,254 | 13,116 | 18,207 | 16,963 |
| Shanxi | 7,254 | 13,116 | 18,207 | 16,963 |
| Shandong | 7,185 | 13,046 | 18,137 | 16,923 |
| Shaanxi | 7,138 | 12,999 | 18,090 | 16,896 |
| Henan | 6,998 | 12,859 | 17,950 | 16,815 |
| Anhui | 7,372 | 13,233 | 18,324 | 17,030 |
| Jiangsu | 7,138 | 12,999 | 18,090 | 16,896 |
| Hubei | 7,301 | 13,163 | 18,253 | 16,990 |
| Sichuan | 7,372 | 13,233 | 18,324 | 17,030 |
| Zhejiang | 7,325 | 13,186 | 18,277 | 17,003 |
| Hunan | 7,138 | 12,999 | 18,090 | 16,896 |
| Jiangxi | 7,325 | 13,186 | 18,277 | 17,003 |
| Yunnan | 7,301 | 13,163 | 18,253 | 16,990 |
| Guizhou | 7,301 | 13,163 | 18,253 | 16,990 |
| Fujian | 7,138 | 12,999 | 18,090 | 16,896 |
| Guangdong | 7,254 | 13,116 | 18,207 | 16,963 |
| Beijing | 7,254 | 13,116 | 18,207 | 16,963 |
| Tianjin | 7,254 | 13,116 | 18,207 | 16,963 |
| Shanghai | 7,254 | 13,116 | 18,207 | 16,963 |
| Chongqing | 7,254 | 13,116 | 18,207 | 16,963 |
| Inner Mongolia | 7,605 | 13,466 | 18,557 | 17,164 |
| Xinjiang | 7,091 | 12,953 | 18,044 | 16,869 |
| Ningxia | 7,091 | 12,953 | 18,044 | 16,869 |
| Tibet | 7,091 | 12,953 | 18,044 | 16,869 |
| Guangxi | 7,185 | 13,046 | 18,137 | 16,923 |
| Qinghai | 7,091 | 12,953 | 18,044 | 16,869 |
| Gansu | 7,138 | 12,999 | 18,090 | 16,896 |
| Hainan | 7,138 | 12,999 | 18,090 | 16,896 |
| National | 7,213 | 13,074 | 18,165 | 16,939 |

Quadrivalent HPV vaccine: Imported Quadrivalent HPV vaccine; 9-valent HPV vaccine : Imported 9-valent HPV vaccine;All costs have been discounted until 2019.
