# Supplementary material for: Human papillomavirus vaccination at the national and provincial levels in China: a cost-effectiveness analysis using the PRIME model
Source: BMC Public Health. 2022 Apr 18;22:777. doi: 10.1186/s12889-022-13056-5 (PMC9014632; doi:10.1186/s12889-022-13056-5)
Supplement: Supplementary file 8 — Additional file 8: Table S17. Cost-effectiveness of the domestic bivalent HPV vaccine; Table S18. Cost-effectiveness of the imported bivalent HPV vaccine; Table S19. Cost-effectiveness of the quadrivalent HPV vaccine; Table S20. Cost-effectiveness of the 9-valent HPV vaccine. [file 12889_2022_13056_MOESM8_ESM.docx]

**Additional file 8. Results of cost-effectiveness analyses of different valence vaccines by province**

**Table S17.** **Cost-effectiveness of domestic bivalent HPV vaccine**

| **Province** | **Domestic bivalent HPV vaccine** | | | |
| --- | --- | --- | --- | --- |
|  | **ICER/GDP per capita** | **＜1 times GDP** | **1~3 times GDP** | **＞3 times GDP** |
| Heilongjiang | 1.34 |  | √ |  |
| Jilin | 1.12 |  | √ |  |
| Liaoning | 0.87 | √ |  |  |
| Hebei | 1.08 |  | √ |  |
| Shanxi | 1.09 |  | √ |  |
| Shandong | 0.70 | √ |  |  |
| Shaanxi | 0.74 | √ |  |  |
| Henan | 0.86 | √ |  |  |
| Anhui | 0.87 | √ |  |  |
| Jiangsu | 0.40 | √ |  |  |
| Hubei | 0.65 | √ |  |  |
| Sichuan | 0.91 | √ |  |  |
| Zhejiang | 0.47 | √ |  |  |
| Hunan | 0.86 | √ |  |  |
| Jiangxi | 0.95 | √ |  |  |
| Yunnan | 1.05 |  | √ |  |
| Guizhou | 1.08 |  | √ |  |
| Fujian | 0.46 | √ |  |  |
| Guangdong | 0.53 | √ |  |  |
| Beijing | 0.30 | √ |  |  |
| Tianjin | 0.55 | √ |  |  |
| Shanghai | 0.32 | √ |  |  |
| Chongqing | 0.66 | √ |  |  |
| Inner Mongolia | 0.77 | √ |  |  |
| Xinjiang | 0.90 | √ |  |  |
| Ningxia | 0.90 | √ |  |  |
| Tibet | 1.00 |  | √ |  |
| Guangxi | 1.15 |  | √ |  |
| Qinghai | 1.00 |  | √ |  |
| Gansu | 1.49 |  | √ |  |
| Hainan | 0.87 | √ |  |  |
| National | 0.70 | √ |  |  |

ICER of each province was compared with GDP per capita of each region, ICER <1 times GDP per capita is very cost effectiveness,1<ICER<3 times per capita GDP has cost effect, there is no cost-effectiveness at ICER>3 times GDP.

**Table S18. Cost-effectiveness of imported bivalent HPV vaccine**

| **Province** | **Imported bivalent HPV vaccine** | | | |
| --- | --- | --- | --- | --- |
|  | **ICER/GDP per capita** | **＜1 times GDP** | **1~3 times GDP** | **＞3 times GDP** |
| Heilongjiang | 2.46 |  | √ |  |
| Jilin | 2.05 |  | √ |  |
| Liaoning | 1.58 |  | √ |  |
| Hebei | 1.95 |  | √ |  |
| Shanxi | 1.98 |  | √ |  |
| Shandong | 1.27 |  | √ |  |
| Shaanxi | 1.35 |  | √ |  |
| Henan | 1.57 |  | √ |  |
| Anhui | 1.56 |  | √ |  |
| Jiangsu | 0.73 | √ |  |  |
| Hubei | 1.17 |  | √ |  |
| Sichuan | 1.64 |  | √ |  |
| Zhejiang | 0.84 | √ |  |  |
| Hunan | 1.56 |  | √ |  |
| Jiangxi | 1.71 |  | √ |  |
| Yunnan | 1.89 |  | √ |  |
| Guizhou | 1.96 |  | √ |  |
| Fujian | 0.84 | √ |  |  |
| Guangdong | 0.96 | √ |  |  |
| Beijing | 0.55 | √ |  |  |
| Tianjin | 1.00 |  | √ |  |
| Shanghai | 0.58 | √ |  |  |
| Chongqing | 1.19 |  | √ |  |
| Inner Mongolia | 1.37 |  | √ |  |
| Xinjiang | 1.65 |  | √ |  |
| Ningxia | 1.65 |  | √ |  |
| Tibet | 1.83 |  | √ |  |
| Guangxi | 2.09 |  | √ |  |
| Qinghai | 1.82 |  | √ |  |
| Gansu | 2.72 |  | √ |  |
| Hainan | 1.59 |  | √ |  |
| National | 1.27 |  | √ |  |

ICER of each province was compared with GDP per capita of each region, ICER <1 times GDP per capita is very cost effectiveness,1<ICER<3 times per capita GDP has cost effect, there is no cost-effectiveness at ICER>3 times GDP.

**Table S19.** **Cost-effectiveness of quadrivalent HPV vaccine**

| **Province** | **Imported quadrivalent HPV vaccine** | | | |
| --- | --- | --- | --- | --- |
|  | **ICER/GDP per capita** | **＜1 times GDP** | **1~3 times GDP** | **＞3 times GDP** |
| Heilongjiang | 3.43 |  |  | √ |
| Jilin | 2.86 |  | √ |  |
| Liaoning | 2.19 |  | √ |  |
| Hebei | 2.71 |  | √ |  |
| Shanxi | 2.75 |  | √ |  |
| Shandong | 1.77 |  | √ |  |
| Shaanxi | 1.87 |  | √ |  |
| Henan | 2.20 |  | √ |  |
| Anhui | 2.16 |  | √ |  |
| Jiangsu | 1.01 |  | √ |  |
| Hubei | 1.63 |  | √ |  |
| Sichuan | 2.27 |  | √ |  |
| Zhejiang | 1.17 |  | √ |  |
| Hunan | 2.17 |  | √ |  |
| Jiangxi | 2.37 |  | √ |  |
| Yunnan | 2.63 |  | √ |  |
| Guizhou | 2.71 |  | √ |  |
| Fujian | 1.16 |  | √ |  |
| Guangdong | 1.33 |  | √ |  |
| Beijing | 0.76 | √ |  |  |
| Tianjin | 1.39 |  | √ |  |
| Shanghai | 0.80 | √ |  |  |
| Chongqing | 1.66 |  | √ |  |
| Inner Mongolia | 1.89 |  | √ |  |
| Xinjiang | 2.29 |  | √ |  |
| Ningxia | 2.30 |  | √ |  |
| Tibet | 2.54 |  | √ |  |
| Guangxi | 2.91 |  | √ |  |
| Qinghai | 2.54 |  | √ |  |
| Gansu | 3.78 |  |  | √ |
| Hainan | 2.21 |  | √ |  |
| National | 1.77 |  | √ |  |

ICER of each province was compared with GDP per capita of each region, ICER <1 times GDP per capita is very cost effectiveness,1<ICER<3 times per capita GDP has cost effect, there is no cost-effectiveness at ICER>3 times GDP.

**Table S20. Cost-effectiveness of imported 9-valent HPV vaccine**

| **Province** | **Imported 9-valent HPV vaccine** | | | |
| --- | --- | --- | --- | --- |
|  | **ICER/GDP per capita** | **＜1 times GDP** | **1~3 times GDP** | **＞3 times GDP** |
| Heilongjiang | 3.21 |  |  | √ |
| Jilin | 2.68 |  | √ |  |
| Liaoning | 2.04 |  | √ |  |
| Hebei | 2.52 |  | √ |  |
| Shanxi | 2.56 |  | √ |  |
| Shandong | 1.65 |  | √ |  |
| Shaanxi | 1.75 |  | √ |  |
| Henan | 2.06 |  | √ |  |
| Anhui | 2.01 |  | √ |  |
| Jiangsu | 0.94 | √ |  |  |
| Hubei | 1.51 |  | √ |  |
| Sichuan | 2.11 |  | √ |  |
| Zhejiang | 1.09 |  | √ |  |
| Hunan | 2.03 |  | √ |  |
| Jiangxi | 2.21 |  | √ |  |
| Yunnan | 2.44 |  | √ |  |
| Guizhou | 2.52 |  | √ |  |
| Fujian | 1.09 |  | √ |  |
| Guangdong | 1.24 |  | √ |  |
| Beijing | 0.71 | √ |  |  |
| Tianjin | 1.30 |  | √ |  |
| Shanghai | 0.74 | √ |  |  |
| Chongqing | 1.54 |  | √ |  |
| Inner Mongolia | 1.74 |  | √ |  |
| Xinjiang | 2.14 |  | √ |  |
| Ningxia | 2.15 |  | √ |  |
| Tibet | 2.38 |  | √ |  |
| Guangxi | 2.72 |  | √ |  |
| Qinghai | 2.38 |  | √ |  |
| Gansu | 3.53 |  |  | √ |
| Hainan | 2.06 |  | √ |  |
| National | 1.65 |  | √ |  |

ICER of each province was compared with GDP per capita of each region, ICER <1 times GDP per capita is very cost effectiveness,1<ICER<3 times per capita GDP has cost effect, there is no cost-effectiveness at ICER>3 times GDP.
