# Supplementary material for: Human papillomavirus vaccination at the national and provincial levels in China: a cost-effectiveness analysis using the PRIME model
Source: BMC Public Health. 2022 Apr 18;22:777. doi: 10.1186/s12889-022-13056-5 (PMC9014632; doi:10.1186/s12889-022-13056-5)
Supplement: Supplementary file 9 — Additional file 9: Table S21. Results of sensitivity analyses. [file 12889_2022_13056_MOESM9_ESM.docx]

| **Index** | **Fluctuation** | **ICER** | | | |
| --- | --- | --- | --- | --- | --- |
|  |  | **Domestic bivalent HPV vaccine** | **Imported bivalent HPV vaccine** | **Quadrivalent HPV vaccine** | **9-valent HPV vaccine** |
| Vaccine price | +20% | 8,770 | 15,804 | 21,913 | 20,435 |
|  | -20% | 5,697 | 10,386 | 14,459 | 13,474 |
| Vaccine delivery cost | +20% | 7,342 | 13,203 | 18,294 | 17,017 |
|  | -20% | 7,125 | 12,986 | 18,077 | 16,892 |
| Cancer treatment cost | +20% | 7,035 | 12,896 | 17,987 | 16,803 |
|  | -20% | 7,432 | 13,293 | 18,384 | 17,106 |
| Target age | 13 | 6,175 | 11,180 | 15,527 | 18,939 |
|  | 14 | 5,947 | 10,767 | 14,953 | 18,239 |
|  | 15 | 5,731 | 10,375 | 14,410 | 17,577 |
|  | 16 | 5,528 | 10,008 | 13,900 | 16,955 |
|  | 17 | 5,335 | 9,659 | 13,415 | 16,364 |
|  | 18 | 5,152 | 9,328 | 12,955 | 15,803 |
|  | 19 | 4,978 | 9,013 | 12,517 | 15,269 |
|  | 20 | 4,812 | 8,712 | 12,100 | 14,760 |
|  | 21 | 4,658 | 8,434 | 11,713 | 14,288 |
|  | 22 | 4,511 | 8,168 | 11,344 | 13,837 |
|  | 23 | 4,371 | 7,913 | 10,990 | 13,405 |
|  | 24 | 4,236 | 7,669 | 10,651 | 12,992 |
|  | 25 | 4,107 | 7,435 | 10,326 | 12,596 |
|  | 26 | 4,002 | 7,241 | 10,055 | 12,264 |
| Coverage rate (3-Dose ) | -10% | 8,147 | 14,660 | 20,316 | 18,923 |
|  | -20% | 9,290 | 16,616 | 22,980 | 21,383 |
| Discount rate | +2% | 18,959 | 33,358 | 45,865 | 35,869 |
|  | -2% | 2,311 | 4,523 | 6,444 | 7,257 |

**Additional file 9.** **Sensitivity analysis results of different indicators**

**Table S21. Results of sensitivity analysis**
